# Supplementary figures and images for: Educating the future generation of researchers: A cross-disciplinary survey of trends in analysis methods
Source: PLoS Biol. 2021 Jul 29;19(7):e3001313. doi: 10.1371/journal.pbio.3001313 (PMC8321514; doi:10.1371/journal.pbio.3001313)

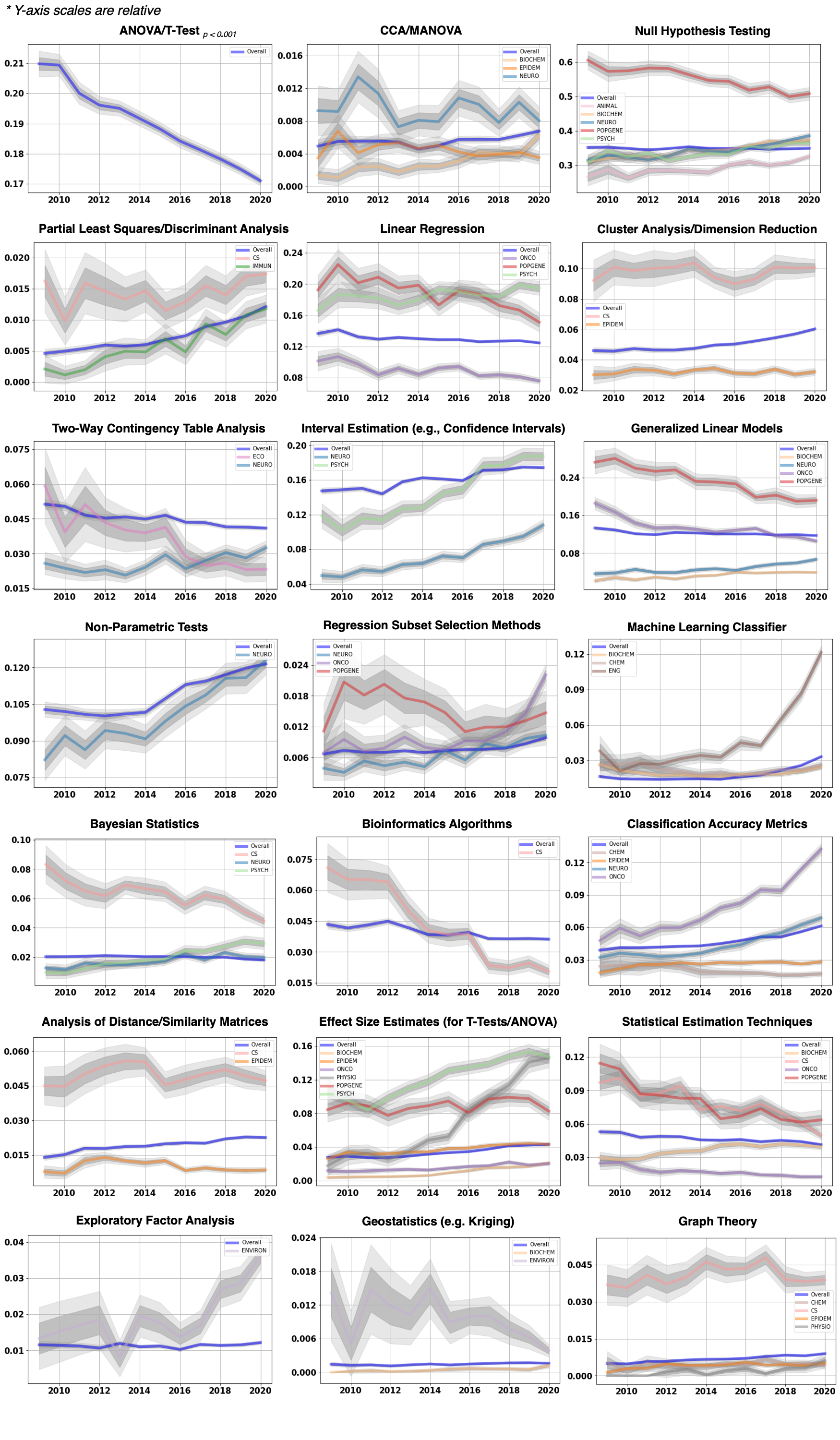

Supplement: S1 Fig — Time series of 21 analytic method categories from 2009 to 2020 (annual frequency). For each analytic method category, the time series represents the proportion of articles that contained a mention of that category in their “Methods/Materials” or “Results” section per year. The time series of each analytic method category is displayed in its own plot with different y-axis scales. Note that because each plot differs in y-axis scale, caution should be observed when comparing trends across categories. Proportion of article counts by year for all analysis methods are provided in S2 Data. Python code for modeling trends of analysis methods is provided at https://github.com/tsb46/stats_history/blob/master/demo.ipynb. (TIFF) [file pbio.3001313.s001.tiff]

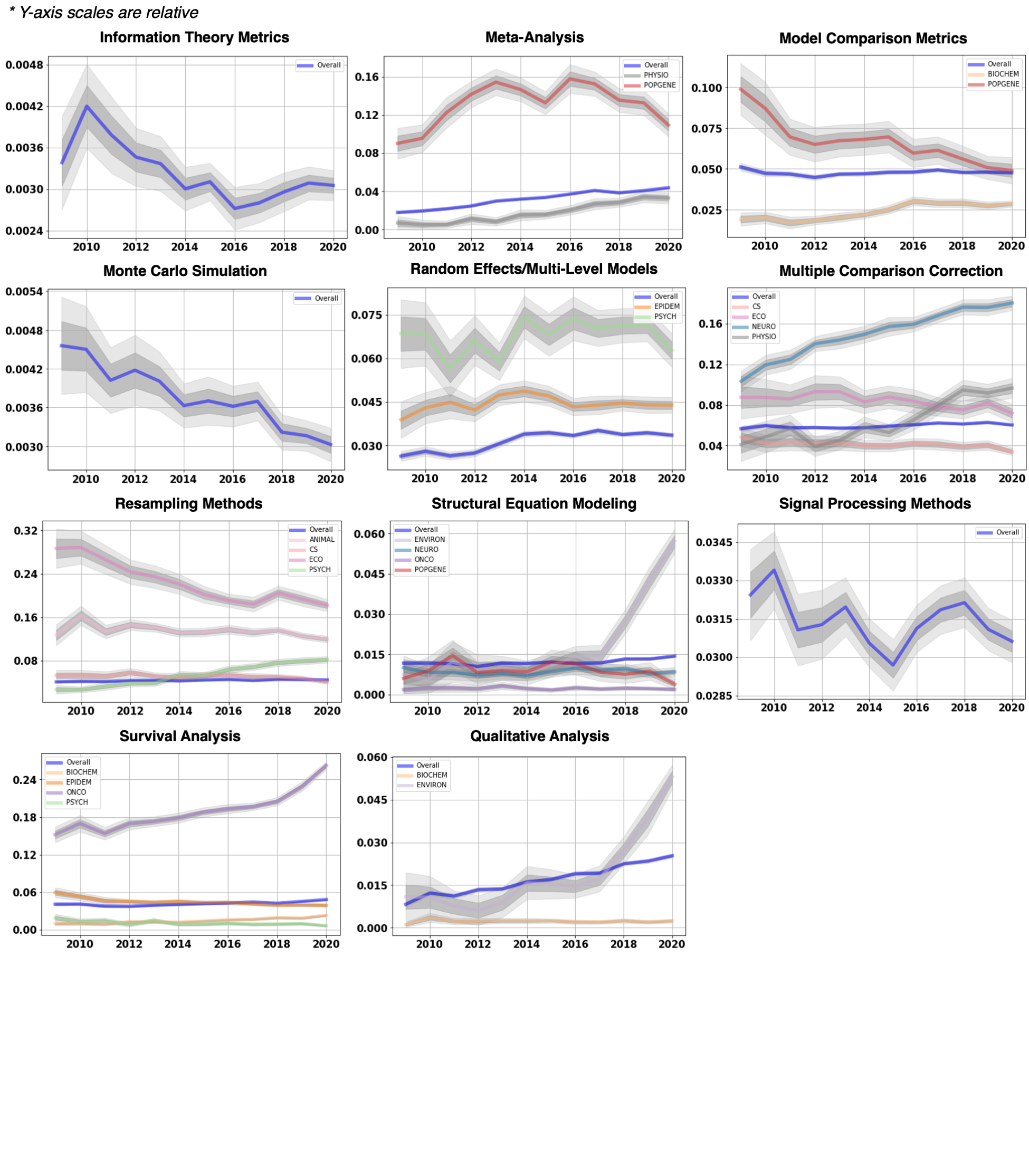

Supplement: S2 Fig — Time series of 11 analytic method categories from 2009 to 2020 (annual frequency). For each analytic method category, the time series represents the proportion of articles that contained a mention of that category in their “Methods/Materials” or “Results” section per year. The time series of each analytic method category is displayed in its own plot with different y-axis scales. Note that because each plot differs in y-axis scale, caution should be observed when comparing trends across categories. Proportion of article counts by year for all analysis methods are provided in S2 Data. Python code for modeling trends of analysis methods is provided at https://github.com/tsb46/stats_history/blob/master/demo.ipynb. (TIFF) [file pbio.3001313.s002.tiff]

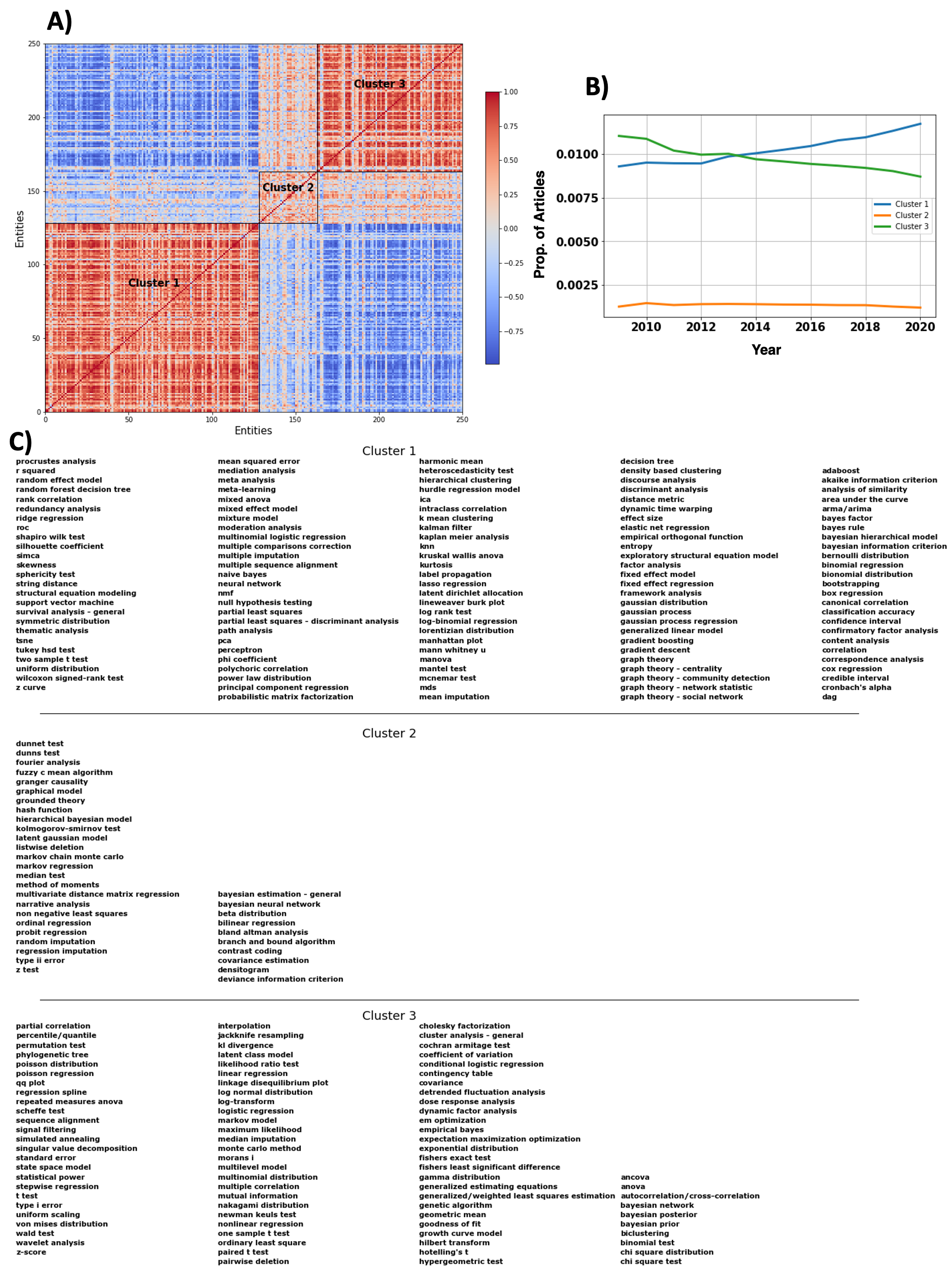

Supplement: S3 Fig — The trends in Figs 3 and 4 display trends in superordinate categories of conceptually similar in analytic methods. For the sake of completeness, we conducted a cluster analysis of individual analytic method trends (N = 250) (before categorization into superordinate categories). Hierarchical clustering was performed to separate 3 clusters of analytic methods with approximately positive (Cluster 1), negative (Cluster 3), and flat (Cluster 2) trends over the study time span. (A) Heatmap of pairwise correlations between analytic method trends sorted according to their cluster assignment. (B) The trends of each cluster over the study time span (2009–2020). (C) The cluster assignments of all analytic methods (N = 250). Cluster assignments for all analysis methods are provided in S5 Data. (TIFF) [file pbio.3001313.s003.tiff]

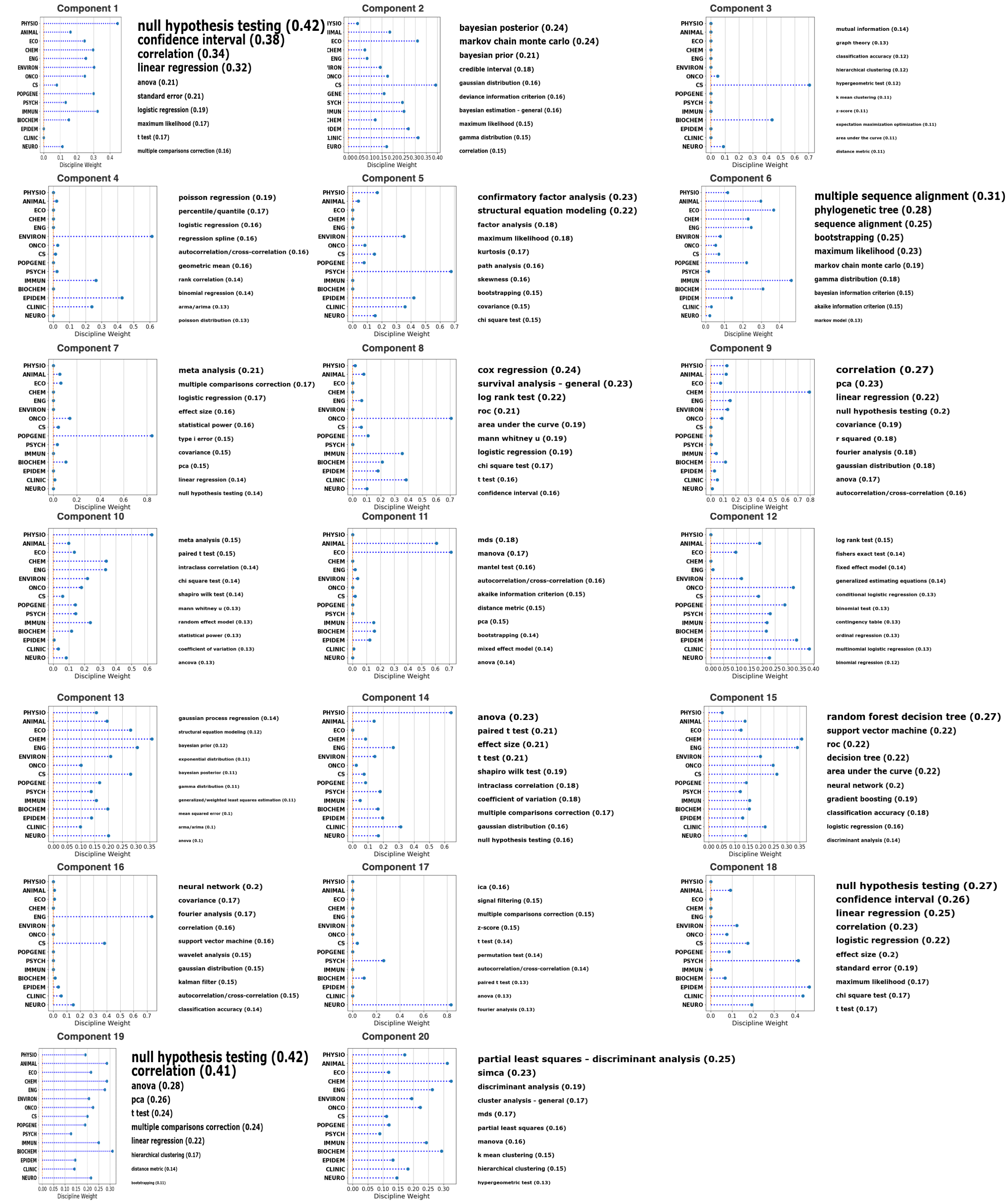

Supplement: S4 Fig — To understand what analytic methods are frequently used together in the same study, we conducted a tensor decomposition of an analytic method co-occurrence by discipline tensor. The tensor decomposition analysis simultaneously models the co-occurrence between analytic methods, as well as their frequency of mentions in each discipline. This figure displays the discipline and analytic method weights from the tensor decomposition analysis. Components from the tensor decomposition are referred to as “method groupings” or groups of analytic methods that frequently occur together in study method and results sections. For each component, or “method grouping,” a stem plot illustrates the weights for each discipline, as well as the top 10 analytic methods, in terms of their weights (sized by their weight). For each component, the discipline weights represent the frequency of usage of that component across each discipline. (TIFF) [file pbio.3001313.s004.tiff]
