# Supplementary material for: Educating the future generation of researchers: A cross-disciplinary survey of trends in analysis methods
Source: PLoS Biol. 2021 Jul 29;19(7):e3001313. doi: 10.1371/journal.pbio.3001313 (PMC8321514; doi:10.1371/journal.pbio.3001313)
Supplement: Attachment — Submitted filename: Reviewer_Response_041221.docx [file pbio.3001313.s010.docx]

April 12, 2021

Dear Dr. Roberts,

Thank you for providing the opportunity for us to revise our submission, now titled *“The changing landscape of analysis methods in the biological and social sciences”*. A summary of major revisions to the manuscript is provided below, followed by a more detailed point-by-point response to reviewers:

**Summary of major changes to manuscript:**

1. In response to multiple reviewer concerns, we have included baseline differences in analytic method mentions as a figure (**Figure 3**) - i.e. all analytic method trends displayed on the same plot and y-axis scale. These baseline differences can be significant, which we acknowledge in our discussion of the results. For example, see pages 7-8.
2. The article corpus has now been expanded in two ways:
   a. Research articles from the year 2020 have now been included in the corpus.
   b. We have expanded our inclusion of section text from research articles. In the previous manuscript, if an article did not include a section title matching phrases expected for a ‘methodology’ section, they were excluded. Reviewers were concerned that this strategy may miss articles with unstandardized methodology section titles, or those without a methodology section in the main text (e.g. in supplementary materials). Thus, we have expanded our list of search phrases to pick up methodology sections with unstandardized titles. Further, we have expanded our section text to include ‘results’ sections of manuscripts as well.
   c. These two changes more than doubled the number of articles in our corpus from 585,362 in the original manuscript, to 1,364,517 in the revised manuscript. All analyses have been updated with this larger corpus, and remain consistent with the findings from our original manuscript.
3. The analysis of analytic method trends have been expanded in two ways:
   a. We have taken a model-based approach to assess the statistical significance of linear increases or decreases in analytic method mentions over the study timespan (2009-2020). Specifically, for each analytic method, we modeled linear trends in the log-odds of an article mentioning that method using a logit link function and a generalized estimating equations (GEE) approach (to account for the nested structure of articles within journals).
   b. In response to reviewer concerns that the ‘overall’ trends displayed may not be representative of every scientific discipline in our corpus, we also included tests of whether individual disciplines significantly deviate from the overall trend.
4. The phrase list used to train the named entity recognition algorithm has been expanded from 700 to 1,129 analytic method phrases. Further, to ensure that our collection of phrases was not limited to the authors’ limited domain-knowledge, we also included analytic methods from the STATO ontology (<http://stato-ontology.org/>), an ontology of statistical methods, in this expanded phrase list.
5. We have included eight supplementary files, figures and tables that provide interested readers with detailed information on all analytic methods discovered from our corpus, article counts by discipline, representative journals by discipline, and additional content from our analyses.
6. The github page for the manuscript (<https://github.com/tsb46/stats_history>) has been finalized. It contains a series of command-line utilities and Python modules for interested readers to replicate the findings in our article, and more importantly, to conduct their own analyses on analytic method usage across the BLS sciences.

We hope you agree that this substantially revised manuscript now meets the high standards for publication in *PLoS Biology*. We look forward to any additional comments, and thank you for your consideration.

Sincerely,

Taylor Bolt, Ph.D.

**Reviewer #1:**

**There are many things to enjoy in the manuscript of Bolt et al., including the scientific motivation, the approach taken, the findings, the writing, and the discussion toward graduate education.**

**Response:** We thank the reviewer for the kind words and insightful comments/suggestions to improve the manuscript.

**In my view there might be few additions that could strengthen the manuscript further with seemingly little additional work.**

**Response:** We thank the reviewer for highlighting areas where the manuscript can be strengthened. We have revised our manuscript in accordance with these comments, and provide point-by-point responses below.

**First, to provide the frequencies of each method for every year in a table as supplemental material.**

**Response:** Following this reviewer’s suggestion, we have now added a new supplementary table summarizing the frequency of mentions for all analytic methods (N=250, **S1 File**).

However, we caution that analyzing the raw frequency counts can be misleading. Counts for all analytic methods are more likely to increase simply due to the increasing number article counts per year in the Pubmed Open Access database. For presentation of analytic method trends in our manuscript, we normalized the counts of each analytic method per year by dividing by the total number of articles per year. These normalized counts represent the proportion of articles mentioning an analytic method per year. As a supplement to the raw frequency table, we also provide the proportion of articles mentioning one analytic method per year in another supplementary table (**S2 File**).

**Second, to include an unbiased analysis of temporal trends. While I anticipate that the examples chosen for Figure 3 and 4 faithfully represent the main observations, they only represent individual methods. Adding an unbiased analysis (e.g.: a clustermap of all pairwise correlations of the frequencies of individual methods) with all methods could help readers to better grasp how prevalent individual temporal trends are, and which methods are more similar (the latter being something that the authors already partially address with Figure 6).**

**Response:** We thank the reviewer for the suggestion to present a more comprehensive summary of individual analytic method trends. We have now computed an analytic method-by-analytic method matrix of pairwise Pearon’s correlation coefficients (215*215 analytic method) that reflects the similarity in yearly trends between each analytic method. This matrix was submitted as input to a hierarchical clustering algorithm (using the Ward linkage criterion). We chose a cluster solution of 3 clusters, as this solution separated out three clusters with a positive, negative and approximately flat trend, respectively. The solution is presented in Supplementary Materials (**S2 Figure**). Consistent with our original conclusions, analytic methods belonging to more ‘traditional’ statistical analysis such as *t*-tests, ANOVAs, linear regression and chi-square tests belong to the emerged cluster that exhibited a linear decrease in prevalence over time, while machine learning/multivariate methods, such as neural networks, support vector machines, graph theory and regularized regression approaches belong to the cluster exhibiting a linear increase over time. Below is the figure:

Supplementary Figure 1. **Data-driven Analysis of Individual Analytic Method Trends.** The trends in **Figure 3** and **Figure 4** display trends in superordinate categories of conceptually similar in analytic methods. For the sake of completeness, we conducted a cluster analysis of individual analytic method trends (N=250) (before categorization into superordinate categories). Hierarchical clustering was performed to separate three clusters of analytic methods with approximately positive (Cluster 1), negative (Cluster 3) and flat (Cluster 2) trends over the study timespan. **A**) Heatmap of pairwise correlations between analytic method trends sorted according to their cluster assignment. **B)** The trends of each cluster over the study timespan (2009-2020). **C**) The cluster assignments of all analytic methods (N=250).

**Third, to spell out the criteria used to define an entity as a method. While it is clear that the authors followed a reasonable rationale, the manuscript implies that only analytical methods relating to statistics, but not other methods such as domain-specific experimental approaches, were considered. If the latter group of methods was included in the training, its absence in the manuscript would warrant a comment on the provided data (see first point) being more extensive, and an explanation for why the manuscript only discussed one set of methods.**

**Response:** We completely agree with the reviewer that the original manuscript was lacking a clear criterion for defining an entity as a ‘research method’. The intended objective of our study was *data analysis methods* - algorithms, statistics or models used to describe, summarize or interpret a sample of data. We excluded from consideration any aspects of research methodology involved in data collection and experimental/study design. We now realize that the original reference to entities as ‘research method entities’ was a potentially confusing phrase to refer to our intended object of study - data analysis methods. We have now replaced ‘research method entity’ with ‘analytic method entity’ to make this more clear. In addition, we have made our criteria for defining an ‘analytic method entity’ more clear in the introduction:

*“Our study aimed to map out the methodological landscape of the BLS disciplines and identify changing trends over the past decade (2009 - 2020). Here we use the term analytic methods to broadly denote any quantitative or qualitative method for data analysis, including any algorithms, statistics or models used to describe, summarize or interpret a sample of data. This definition is meant to exclude those elements of research methodology involved in data collection, or experimental or study design.”* (Pg. 2)

**Other:**

**References to Fig 1A and Fig 1B within the text seem to point to the wrong figure.**

**Response:** We thank the reviewer for noticing this mistake. These references have now been amended (Figure 2A and Figure 2B).

**The criteria for classifying a method into "traditional" or "advanced" could be stated out.**

**Response:** We thank the reviewer for the opportunity to clarify our classification of methods in the original manuscript. As described in our response to the next comment, we have moved away from a potentially controversial distinction between ‘traditional’ and ‘advanced’ analytic methods to a more nuanced classification scheme of analytic methods.

**It is not clear why the authors chose "traditional" and "advanced". While I do not wish to impose my view on the manuscript, some clarifying words might be helpful. For instance, I would have regarded "PCA" as a "classical" method since it ultimately relates to variances and Euclidean spaces. Similarly, I would have seen "effect size" and "confidence interval" outside of "traditional" (even if not "advanced") as they would better align with non-parametric tests and data that does not follow a narrow set of characteristics that would allow the appropriate usage of t-tests or ANOVA etc.; Similarly, I would anticipate "effect size" and "confidence interval" to align with "big" data, where the size of the dataset would avoid the need to use some other "traditional" approaches to estimate the spread of the data.**

**Response:** We agree with the reviewer that distinction between ‘traditional’ and ‘advanced’ in the original manuscript was insufficient and perhaps misleading . We have decided to not impose this rough distinction on any of the methods we describe in the revised manuscript. Further, the separation between these two ‘types’ of methods has been removed from **Figure 2**. Rather, each method is now treated individually. We now refer to these two sets of analytic methods as those ‘commonly taught in introductory research methods and statistics courses’ and those ‘taught in more advanced statistics and computer science courses’, respectively. This distinction emphasizes an educational distinction, as opposed to fundamental mathematical or conceptual distinctions between the methods. At times, for brevity, we do refer to these two sets of methods as ‘introductory’ or ‘advanced’ methods, but caution the reader that these are merely distinctions denoting typical educational sequences, as opposed to some more fundamental mathematical or conceptual underpinning. Further, we provide the following cautionary note in the introduction of these two ‘types’ of methods:

*“We make a distinction between those analytic method categories that are commonly taught in introductory research method and statistics courses - e.g. null-hypothesis testing, t-tests/ANOVA, linear regression, two-way contingency tables (e.g. chi square test), and interval estimation (e.g. confidence intervals)* [*^20,21,21^*](https://www.zotero.org/google-docs/?9sSkKx)*, and those analytic method categories taught in advanced statistics or computer science courses - e.g. dimension reduction and clustering techniques (e.g principal component analysis (PCA), non-negative matrix factorization (NMF), K-means clustering), machine learning classifiers (e.g. support vector machines, random forest classifier), and regression subset selection methods (e.g. Lasso regression). We nominally refer to these two groups as ‘introductory’ and ‘advanced’ analytic methods, respectively. Note, we do not intend by this distinction to mean that ‘introductory’ methods are less sophisticated or appropriate for data analysis than ‘advanced’ analytic methods. This distinction is merely meant to distinguish between those analytic methods typically taught in introductory courses in data analysis from more advanced undergraduate and graduate data analysis courses.*” (Pg. 7)

**Possibly already addressed by my first main point, I would be curious on the temporal trends of those special cases of methods that can be used to evaluate whether other methods are suitable (e.g.: Lilliefors).**

**Response:** We have provided all analytic method frequencies and proportions in our corpus in the Supplementary Material (**S1 File** and **S2 File**). We did not find the example method cited (‘Lilliefors’) by the reviewer in a search through the analytic methods found by our named entity recognition algorithm. However, we do have in our corpus various model assumption tests and graphics - normality tests (e.g., Shapiro/Wilk Test, qq-plot),

**Providing the accuracy of the classification of disciplines on a per-discipline level, rather than only in aggregate, could assist people from smaller disciplines to better assess the strength of the findings of this manuscript within their own discipline.**

Response: We thank the author for this suggestion.We have now included individual discipline accuracy levels in the Methods and Materials section:

*“The final MNB model achieved an accuracy of 74.9%. The accuracy achieved for each individual discipline are as follows: ANIMAL – 71.2%, BIOCHEM – 72.2%, CHEM – 88.4%, CLINIC – 77.6%, CS – 79.2%, ECO – 75.3%, ENG – 67.4%, ENVIRON – 67.8%, EPIDEM – 65.6%, IMMUN – 64.0%, NEURO – 87.1%, ONCO – 84.5%, PHYSIO – 69.6%, POPGENE - 60.0%, and PSYCH – 69.2%.”*

**It is unclear how the classification of disciplines would be affected by articles that do not fall into any of the 15 chosen classes - or if this could be a problem that could likely be safely ignored (e.g.: if only applicable to few publications).**

Response: We appreciate this concern. Our classification of disciplines was largely based on pragmatism. As we note in the text, it is unlikely that these disciplinary categories represent mutually exclusive categories of disciplines, or would suffice as a useful ontology of scientific disciplines in Pubmed. Rather, an informal survey of the corpus led us to choose the categories to balance precision with sufficient sample size for each category. There will undoubtedly be articles that do not squarely fit within one of these 15 categories, or overlap with several. Further, we acknowledge that there will be discipline categories that are not represented in the Pubmed Open Access subset, such as the discipline of economics, most of experimental and theoretical physics, astronomy, and geology, to name a few. For those readers interested in testing different categorizations of the Pubmed Open Access subset, we provide a command-line interface tool (https://github.com/tsb46/stats_history) to train our classification model with different categorizations of methods. We hope that this tool will be helpful for future applications in this space. We encourage the interested user to modify this tool for their own research purposes.

**Reviewer #2:**

**Review report for "Historical and cross-disciplinary trends in the biological and social sciences reveal an accelerating adoption of advanced analytics" by Bolt et al.**

**This paper explores the cross-disciplinary usage of different research methods by analyzing a large corpus of research publications largely in biological and social science fields. Specifically, the authors leveraged data-driven text-mining tools to identify research methods from publication texts and then analyzed the cross-disciplinary trends over the past 10 years. The authors reported several major findings: (1) The classical statistics and regression-based methods have declined in the past decade, while machine-learning approaches have seen a significant increase. (2) Research method categories are uniquely associated with each research discipline. (3) There are co-occurrence patterns in research method usage, where research groupings can be further decomposed into cross-discipline and within-discipline method categories. Lastly, the authors suggested some policy implications of their findings, for example, on cross-disciplinary collaboration, training of researchers,**

**and education in research methods.**

**Response:** We appreciate this concise summary.

**The paper considers an interesting research question and reveals an important trend in research disciplines, showing the ongoing methodological shifts in a variety of scientific communities. In particular, as the development of novel research methods, computational tools, and large-scale datasets, there may be substantial shifts in the methodology and framework of doing research in many traditional fields. For example, the advances of deep learning in recent years have not only promoted the rapid development of computer science fields but also remarkably benefited other research fields in prediction and computation. Here, the Alpha Fold can be a nice example (Improved protein structure prediction using potentials from deep learning. Nature 577, no. 7792 (2020): 706-710). In this sense, I think this paper has its merit in providing some initial evidence on the adoption of advanced analytics across several fields.**

Response: We thank the reviewer for this insight, and appreciate the suggestion to include the Alpha Fold model as a nice example of a deep learning application in biology. Which we have integrated in the revised manuscript as follows:

*“Increasingly, analytic methods developed in one discipline find fruitful application in another. For example, deep-learning, a machine learning technique developed by the artificial intelligence community, has successfully been used by biologists to predict three-dimensional protein structure*[*^14^*](https://www.zotero.org/google-docs/?0wbyLT)*. The explosive adoption of neural networks across biology and multiple other fields illustrates the need for educational training to note these trends and keep pace with the demand for expertise in these emerging advanced analytic approaches.”* (Pg. 2)

**However, in my view, this paper still has room to improve before its consideration for publication. In the following, I would like to provide some comments and suggestions, which may be helpful for the authors to further strengthen their paper.**

Response: We thank the reviewer for constructive suggestions.

**The authors observed that the usage of some traditional research methods (such as t-test, ANOVA, and regressions) has declined in the publications over the considered period, while the advanced analytics (such as machine learning methods) have seen an increase during the same period. The results are seeming obvious and the interpretation of these major observations remain largely limited in this paper. First, what are the driving forces behind these observations? These could be due to the publication landscape shifts towards computing and statistical fields, the changes of research tasks of traditional fields, and the availability of new types of data sources. For example, the use of LandScan satellite image data in the study of social and economic status inference requires image processing methods other than traditional regression methods based on survey and census data [Combining satellite imagery and machine learning to predict poverty. Science 353, no. 6301 (2016):**

**790-794].**

**Response:** We are grateful to the reviewer for drawing our attention to this important consideration.

The goal of our study was to document temporal and cross-disciplinary trends over the past decade. Due to the exploratory nature of our analysis, our ability to examine the potential driving forces behind these changes is limited. However, there are several explanations that we cautiously propose as potential explanatory factors in the revised discussion:

“This study made no attempt to examine the potential causal factors behind these trends, but we hypothesize that they could be due to several reasons: 1) the collection of larger and more complex datasets, 2) the recent popularity of data science as a tool in academia and industry, or 3) an increasing realization among researchers that manuscripts containing advanced analytics are more likely to impress reviewers and editors.”

**Second, what was the baseline to understand the relative share or the adoption of research methods across different methods? By definition, some research methods and tools are old and traditional, and some others are new and advanced. Even for new and advanced general-purpose methods/technologies, it takes some time for them to be used by many fields. Also, they will be replaced by more recent ones as technology advances. It is not clear enough how to compare the observed patterns with a baseline.**

**Response:** We thank the reviewer for bringing up this critical point. To clarify,the trends reported in the original manuscript were *relative* trends, and difficult to compare across time-series because the original plots had different y-axis scales. We have now provided a new figure (**Figure 3**) that provides all time-series on the same plot that illustrates differences in baselines between method mentions. As the figure illustrates, baseline differences are indeed very prominent between methods. We also have emphasized these baseline differences in the revised text, for example:

*“As can be observed from* ***Figure 3****, introductory statistical methods - e.g. null-hypothesis, testing, t-tests/ANOVA, linear regression, confidence intervals - have remained the dominant analytic methods mentioned in BLS methodology and results sections over the past decade. Null-hypothesis testing concepts and statistics (e.g. p-values, null hypothesis, and alternative hypothesis) are by far the most commonly mentioned analytic ‘methods’ in the BLS sciences - approximately 35% of articles per year mention one or more analytic methods in this category.”* (Pg. 7).

*“We find that the analytic methods commonly taught in introductory research methods and statistics courses remain the most commonly mentioned methods in research article ‘Methods’ and ‘Results’ sections. However, while dominant, these methods have largely declined in prominence, or remained stable, over the timespan of the study (2009-2020).”* (Pg. 15)

**Third, related to the first point, advanced analytics have been increasingly used, but it is not clear if they are used to address the same research tasks or new research tasks in one research field. In my view, without these further explorations and in-depth understanding of these observations, the contributions of this paper are best limited.**

**Response:** We appreciate this astute observation. Our goal in the current study was to make first steps towards a “bird’s-eye view” of data analytic trends across the biological and social sciences. As the reviewer notes, this analysis does not disentangle whether advanced analytics are increasingly being applied to the same or to new research questions within a given field. It is not immediately clear what approach one would take to shed light on this challenging question. As an example, in cognitive neuroscience (a rapidly growing subfield within psychology), the use of mass univariate approaches for the statistical analysis of neuroimaging data is still commonplace despite the increasing adoption of multivariate analyses in this field. Still, one approach has not entirely replaced the other for localizing brian activity, and in fact each approach provides complementary information regarding aspects of brain function. For this reason, most human neuroimaging papers reporting multivariate results include univariate results as well. This example is meant to illustrate that as different disciplines are associated with different explanatory goals, it would be difficult to ascertain what constitutes the same or different research questions in a given field without more in-depth knowledge of the open questions and assumptions within each field. We acknowledge that a future direction along the lines of what the reviewer suggests is warranted, and that further research would need to be conducted for each discipline with the aid of experts in those fields.

**This paper relied on the entity recognition of research methods based on the description of Methods and Materials (method, material, measure, analysis, and statistical) in the publication texts. The authors first developed an initial list of 700 phrases that are commonly used research methods. Then, they applied a statistical named entity recognition algorithm to the entire corpus of methodology section texts to generate the final list of detected research method entities. Finally, they pre-processed the list of method entities and resulted in 1,218 final method entity vocabulary. There are several potential issues on the method. First, not all papers have the specific section of Methods and Materials. For many journals in social and economic journals, the method, material, measure, analysis, and statistical sections are also not standardized.**

Response: We appreciate this critical point. It is indeed the case that some journals do not have standardized section titles, and some journals limit methods and materials to supplementary materials. Following the suggestion of the reviewer, we have expanded our list of potential search phrases to identify ‘methods sections’. More importantly, in response to the reviewers comments, we have included ‘Results’ section texts as an additional source of information for an article's method usage. We reason that if a given analytic approach appears in a ‘Results’ section of a manuscript, it was likely utilized in data analysis. Inclusion of terms from ‘Results’ sections should mitigate the concern regarding journals that do not contain standard ‘Methods’ sections.

-> hmm, hasn’t the tagging been done by the government paid pubmed team by hand? If yes, we could mention this in an additional 2-3 sentences, saying that this follows an established procedure that is out of our reach, here we are building on these giants’ shoulders...

**It is not clear how the selection of the body of full text affect the results.**

**Response:** We appreciate this concern. We have opted to not include the full-text of articles due to the fact that analytic methods may be referred to in the introduction and/or discussion as a comparison to the methods they used, or to cite the use of a method in previous research. Restricting our analyses to text found in ‘Methods’ and ‘Results’ sections mitigates this concern . We acknowledge that it is still the case that mentions within these sections of texts are not unequivocal evidence of their usage. See our comment below.

**Also, the mention of research method entity does not mean the use of them. For example, the Methods section of this paper (which includes many subsections, pages 13-18) mentioned a lot of research method entities, however, not all of them are used in this paper (If the authors apply their method to this paper, what can be extracted and identified?). The use of full text of Methods and Materials provides more information, while, at the same time, this may also introduce as much noises.**

**Response:** We agree with the reviewer that mentions of analytic methods in ‘Methods’ and ‘Results’ sections are not unequivocal evidence of their usage in the paper. Our restriction of article text to these sections was intended to mitigate some of these false-positives. Of course, it is still possible that some entities identified by the NER algorithm were not actually used in the paper. However, due to the large-scale nature of the analysis and the size of the corpus (~1.3 million articles) it would be infeasible to spot-check all articles manually. We have now emphasized this difficulty in the newly added limitations section in response to this helpful reviewer comment:

*“It should be noted that the corpus and methods used in this analysis are limited in many respects. First and foremost, our study relies on mentions of an analytic method in a research article as an indicator for the usage of that analytic method in the article. However, the mention of an analytic method is not an unequivocal indication of its usage for data analysis in a research article. Therefore, we restricted our analysis of research articles to their ‘Methods’ and ‘Results’ sections, where mentions of an analytic method should be more closely associated with their usage. However, due to the infeasibility of spot-checking a large corpus (~1.3 million articles) manually, there may be cases of analytic method mentions that did not imply their usage in our sample.”* (Pg. 16-17)

**Second, the recognition of research method entity is lack of independent validations. The used statistical named entity recognition algorithm and the convolutional neural network under the SpaCy package seem to be a black box. The 16,020 research method entities discovered by the algorithms could be very messy, and the resulting 1,218 final method entities seem to be only a very small portion (less than 10%). The used method and the list are lack of transparency. It remains unclear how the selection of entities affects the results, how to**

**validate the recognized entries, and whether they are meaningful.**

Response: We thank the reviewer for providing the opportunity for us to clarify our approach. We recognize the concern that some entities identified by the NER [unpack] could be nonsense phrases or phrases not referring to analytic methods. To address this, our final preprocessing step included an entity disambiguation stage, where the authors manually reviewed each entity string to verify its veracity, and categorized together entities referring to the same method with different phrases.

More generally, the Pubmed database is a unique resource of its kind, which creates tremendous opportunity, but unfortunately also precludes obvious validation in other research article databases.

**Third, the author used data from Pubmed database, which covers mainly biological and social science fields. Meanwhile, the authors only included a random subset of Plos One articles in the corpus. It remains unclear how the sampling strategies affect the results. For example, whether the results are robust if using the full sample of publications such as Microsoft Academic Graph (MAG) data and Web of Science (WoS) data.**

**Response:** We appreciate this point regarding databases included in the current analysis. The original random sampling strategy to reduce the number of PLOS ONE articles was implemented to account for the outsized number of articles from this journal in the corpus. We can confirm, as noted in the original manuscript, that this sampling strategy had no effect on the primary results of the paper. However, to avoid criticism that such a subsetting strategy unfairly singles out PLOS One articles for removal, we have included all PLOS One articles in the analyses of the current revision of the manuscript.

We agree with the reviewer that it is unclear how these results would generalize to article databases based on diverging approaches to article taxonomies, or a more varied assortment of scientific disciplines. Unfortunately, both Microsoft Academic Graph and Web of Science only includes article metadata, as opposed to an article’s full-text - a key ingredient of the present investigation. Thus, we are unable to apply the same sort of analyses applied to the PMC Open Access Subset, to these journal meta-data sources.

However, it is important to note that these results may not generalize to scientific disciplines outside of those in the PMC Open Access subset. As we note in the limitations section:

*“Fourth, some scientific disciplines may be less well represented in this survey, including experimental and theoretical physics, anthropology, astronomy, cosmology, economics, sociology, and geology. Future studies with a more comprehensive corpus of scientific publications will provide deeper insight into the historical and cross-disciplinary trends in scientific data analysis.”* (Pg. 17)

**Fourth, the authors used a machine-learning text classification approach to classify each single article into a set of disciplines. The method is not clearly presented, and it remains unclear whether the identified fields are well aligned with the MAG fields or WoS fields. Additional independent validations are necessary.**

Response: We realize that we have not been sufficiently clear in the original manuscript. To clarify, no attempt was made to match our discipline categorization with the Microsoft Academic Graph (MAG) or Web of Science (WoS) fields. We make no claim that this categorization of disciplines represents an ontology for future bibliometric work. As we note in the main text, the categorization of disciplines for our analysis was purely pragmatic, so as to balance the sample size of articles across potential disciplinary categories. This categorization was conducted through an informal survey of a large number of articles in the corpus. A high-resolution ontology, such as that provided by MAG or WoS would have been less useful for this purpose, which was to assess broad disciplinary trends

**Specific comments:**

**1. In the abstract section, the authors presented "At the same time, there is increasing concern that education in quantitative methods is failing to adequately prepare students for contemporary research. These trends have led to calls for educational reform to undergraduate and graduate quantitative research method curricula". However, I failed to find evidence or references in the introduction section that can support these claims. In my view, these sentences would be better to be placed in the discussion section or relocated to (after a revision) the end of the abstract as the implications of this paper.**

**Response:** We appreciate this suggestion, and have now included an updated list of references in the introduction to support our claims in the abstract:

*“Accelerated by trends in open-source science in the past decade, the methodological landscape of the biomedical, life and social (BLS) sciences is becoming increasingly complex. The classic statistical tools (e.g. T-tests, analysis of variance, linear regression) taught in introductory statistics courses are, at times, perceived insufficient to prepare researchers for the age of big data, machine learning, and open source software. Concerned that BLS sciences educational training is struggling to keep up with these trends, many researchers and statisticians have advocated for educational reform to introductory research methods and statistics courses ^8-13^.”* (Pg. 2)

Below are the references cited:

*8*[*.	Ridgway, J. Implications of the Data Revolution for Statistics Education. Int. Stat. Rev.* ***84****, 528–549 (2016).*](https://www.zotero.org/google-docs/?EzvBfI)

*9*[*.	Cobb, G. Mere Renovation is Too Little Too Late: We Need to Rethink our Undergraduate Curriculum from the Ground Up. Am. Stat.* ***69****, 266–282 (2015).*](https://www.zotero.org/google-docs/?EzvBfI)

*10*[*.	Gibson, E. W. Leadership in Statistics: Increasing Our Value and Visibility. Am. Stat.* ***73****, 109–116 (2019).*](https://www.zotero.org/google-docs/?EzvBfI)

*11*[*.	Diggle, P. J. Statistics: a data science for the 21st century. J. R. Stat. Soc. Ser. A Stat. Soc.* ***178****, 793–813 (2015).*](https://www.zotero.org/google-docs/?EzvBfI)

*12*[*.	Akil, H. et al. Neuroscience Training for the 21st Century. Neuron* ***90****, 917–926 (2016).*](https://www.zotero.org/google-docs/?EzvBfI)

*13*[*.	Baumer, B. A Data Science Course for Undergraduates: Thinking With Data. Am. Stat.* ***69****, 334–342 (2015).*](https://www.zotero.org/google-docs/?EzvBfI)

**2. In the introduction section, it would be better to add some discussions on the motivation of this paper. In particular, it would be nice if the author can develop some hypotheses in this section and then test them using data. In other words, what would the readers anticipate after reading the introduction could be better covered. In addition, some references can be used to better support the authors' narrative, for example, the open science in the past decade [Foster, E. D., & Deardorff, A. (2017). Open science framework (OSF). Journal of the Medical Library Association: JMLA, 105(2), 203], and the use of large-scale data and interdisciplinary methods in social and economic fields [Computational socioeconomics. Physics Reports 817 (2019): 1-104]. Moreover, the core results of this paper could be embedded in the introduction section. The current introduction was too brief in my view.**

Response: We appreciate the suggestion to provide more background in the introduction. As suggested, we have now expanded the introduction in response to the reviewer’s concerns. First, we have included more references to support our narrative. For example:

*“The methodological landscape of the biomedical, life and social (BLS) sciences is becoming increasingly complex. This increasing complexity is largely driven by the advent of open-source science* [*^1^*](https://www.zotero.org/google-docs/?uQX45j)*, the availability of large, complex datasets* [*^2–4^*](https://www.zotero.org/google-docs/?G0mSC4)*, and increasing computational resources* [*^5–7^*](https://www.zotero.org/google-docs/?ILfnCu) *.”* (Pg. 2)

Below are the references:

[*1.	Foster, E. D. & Deardorff, A. Open Science Framework (OSF). J. Med. Libr. Assoc. JMLA* ***105****, 203–206 (2017).*](https://www.zotero.org/google-docs/?EzvBfI)

[*2.	Bzdok, D. & Yeo, B. T. T. Inference in the age of big data: Future perspectives on neuroscience. NeuroImage* ***155****, 549–564 (2017).*](https://www.zotero.org/google-docs/?EzvBfI)

[*3.	Fan, J., Han, F. & Liu, H. Challenges of Big Data analysis. Natl. Sci. Rev.* ***1****, 293–314 (2014).*](https://www.zotero.org/google-docs/?EzvBfI)

[*4.	Marx, V. The big challenges of big data. Nature* ***498****, 255–260 (2013).*](https://www.zotero.org/google-docs/?EzvBfI)

[*5.	Gao, J., Zhang, Y.-C. & Zhou, T. Computational socioeconomics. Phys. Rep.* ***817****, 1–104 (2019).*](https://www.zotero.org/google-docs/?EzvBfI)

[*6.	Efron, B. & Hastie, T. Computer Age Statistical Inference. (Cambridge University Press, 2016).*](https://www.zotero.org/google-docs/?EzvBfI)

[*7.	Hanson, B., Sugden, A. & Alberts, B. Making Data Maximally Available. Science* ***331****, 649–649*](https://www.zotero.org/google-docs/?EzvBfI)

We have also included the core results our paper in the introduction section:

*“Our survey found that analytic methods commonly taught in introductory research methods and statistics courses (eg. t-test, ANOVA) remain the most commonly mentioned methods in BLS research articles over the past decade. However, these methods have largely declined in prominence, or remained stable, over the past decade. On the other hand, multivariate statistics and machine learning methods have exhibited a consistent, sometimes exponential, increase in mentions from 2009 to 2020. Further, we found that analytic methods are not equally distributed across BLS disciplines, but tend to cluster into certain disciplines over others.”* (Pg. 2-3)

**3. In the pre-processing workflow of research method entity, the granularity of the research entity should be justified. It seems that "statistically significant" is the same as "statistical significance", but they are two method entities in this paper (on page 4). Some methods are essentially the same but named differently, for example, Gaussian Process regression and kriging in geostatistics. How will these issues affect the results?**

**Response: We appreciate this concern.** We have now conducted a more thorough review of analytic method entities in the ‘entity disambiguation’ phase of the preprocessing pipeline. Separate entities (i.e. phrases) that refer to the same analytic method have now been categorized into the same method. For example, ‘statistical significance’ and ‘statistically significant’ now refer to the same analytic method in our analyses - null-hypothesis testing (or statistical significance testing).

**In addition, the 15 research disciplines of publications should be cross-validated. Response: As suggested,** we have now included cross-validation statistics from our discipline-classification algorithm in the methodology section:

*“The final MNB model for training involved 15,000 features, 15 discipline categories to be predicted, and 1,470 training samples. We assessed the model accuracy using a repeated K-fold cross-validation approach (#folds = 10, # of repeats = 10). The final MNB model achieved an accuracy of 74.9%. The accuracy achieved for each individual discipline are as follows: ANIMAL – 71.2%, BIOCHEM – 72.2%, CHEM – 88.4%, CLINIC – 77.6%, CS – 79.2%, ECO – 75.3%, ENG – 67.4%, ENVIRON – 67.8%, EPIDEM – 65.6%, IMMUN – 64.0%, NEURO – 87.1%, ONCO – 84.5%, PHYSIO – 69.6%, POPGENE - 60.0%, and PSYCH – 69.2%.”*

**The relationship between the classification of research fields and the grouping of method entities is not clear. It seems not necessary that "the pre-processed method entities before the entity disambiguation step were supplied to a third analytic pipeline".**

**Response:** We thank the reviewer for noting this ambiguity in the description of the pre-processing pipeline. The reviewer is correct that there was no necessary connection between the grouping or classification of analytic method entities and the classification of articles. The cited sentence from the original manuscript gave the appearance that the entity disambiguation phase was a necessary step before article classification. Article classification was a separate component of the pipeline that did not rely on output from the NER algorithm. It simply used the abstract, article tile and journal title text as input. Thus, we’ve removed references to article classification as a step in the entity preprocessing pipeline. Further, we’ve changed the arrows in the figure of the preprocessing pipeline (**Figure 1**) to emphasize there is no connection between the NER algorithm and article classification (it originally had an arrow going from the NER step to the article classification step):

**In addition, one paper can still belong to multiple research disciplines, correct? If one paper used Method A for 10 times and Method B for 1 time in a paper, does the entity recognition algorithm count them once each or different times?**

**Response: We thank the reviewer for providing the opportunity for us to clarify this important point.** We did not count an analytic method more than once if it appeared in the same article. We argue that the number of references to the same analytic method in a paper does not provide evidence for the frequency of its usage in the paper. Thus, analytic method counts in our analyses were binarized - 0: did not appear in the article, or 1 - did appear in the article. While it is indeed the case that one paper can belong to multiple research disciplines, for simplicity here each paper was categorized into the dominant discipline. We have included this simplification as a limitation in the discussion section:

*“Fifth, our classification of scientific disciplines required that an article is assigned to one, and only one discipline. While the majority of research articles in our corpus may fit into one scientific discipline over others, some will be multidisciplinary, particularly for those disciplines with similar research agendas and regular collaboration (e.g. oncology, clinical research and immunology).”* (Pg. 17).

**4. On page 4 of this paper, the "Figure 1A" and "Figure 1B" should all be "Figure 2A" and "Figure 2B". Moreover, it is not clear how to interpret Figure 2B. The size of nodes shows the Discipline Article Counts, but they are not in the correct size.**

**Response:** We thank the author for noting this mistake in the original manuscript, the figure references have now been corrected and the size of the nodes in Figure 2B.

**The authors presented that "This approach made apparent that four disciplines stood out as relative outliers in research method usage: Oncology, Population Genetics, Evolution/Ecology and Chemistry/Material Sciences. As we observe below, among all 15 candidates, these select disciplines revealed a unique profile of research method usage". However, from Figure 2B, I could not find these observations, for example, there seems no evidence showing "four disciplines stood out as relative outliers".**

**Response:** To clarify, the original identification of outliers was based solely on visual inspection of the two-dimensional embedding plot in Figure 2B. We have now identified Chemistry/Material sciences and Evolution/Ecology as outliers through both visual inspection and the Euclidean distance between each discipline’s position in the embedding space and the centroid position (i.e. average of all discipline’s position). Consistent with visual observation, the two disciplines with notably greater Euclidean distance from the average position (centroid) are Chemistry and Evolution/Ecology. The steps of this outlier calculation can be seen in our demo notebook (Sec 1.2.3) for the paper (<https://github.com/tsb46/stats_history/blob/master/demo.ipynb>).

**In the caption of Figure 2, the author presented "The research disciplines with the highest article counts were primarily biomedical and clinical disciplines". Yet, the evidence is also missing.**

**Response:** We thank the author for bringing this to our attention. The number of article counts in Figure 2 were illustrated by the size of the discipline nodes in Figure 2B as indicated in the legend to the right of the plot (i.e. the size of the node was proportional to the number of article counts in each discipline). We have enlarged the size of the nodes in Figure 2B to better illustrate our claims.

**5. At the top of page 6, the authors presented that "Interestingly, the only classical statistical methods that we observed to gain in adoption over the past decade are effect sizes and confidence intervals. The increasing adoption of effect sizes and confidence intervals may reflect the increased pressure from institutions and researchers (10-12) to report effect sizes and confidence intervals along with significance tests in peer-reviewed research". In my view, the reason behind the increasing adoption of effect sizes remains unclear. I am wondering if the authors could provide some evidence to support these claims.**

**Response:** We thank the author for bringing this to our attention. In the revision version of the manuscript, we focused our commentary on confidence intervals, due to the uncertainty over what exactly constitutes an ‘effect size’ in most statistical analyses. We have added a sentence in our discussion of confidence intervals to explain our reasoning behind the original statement:

*“Interestingly, interval estimation methods, such as confidence intervals, have exhibited an increase in mentions over the past decade, perhaps reflecting the increased pressure from institutions and researchers (10–12) to report confidence intervals along with statistical significance tests and p-values in peer-reviewed research. As opposed to p-values, confidence intervals have the advantage of providing information regarding both the size and uncertainty of a point estimate.”*

**6. In Figure 3, the authors used different scales in the y-axis for research disciplinary categories. Yet, it is very hard to make the comparison across different categories. It would be also nice to show the figure that used 0 as the starting point for each panel, for example, in the online supplementary information. Moreover, there could be field differences, for example, the uses of neural networks are different in the computer science field and in the social science field. It would be interesting to explore more and include these new results in the supplementary information.**

**Response:** We thank the reviewer for this suggestion. The original presentation of analytic method trends in Figure 3 was meant to illustrate the relative trends across the study timespan. This made comparison across analytic methods trends difficult. To respond to this reviewer point, we have included a new figure (**Figure 3**) with all analytic method trends displayed in the same plot, allowing for easier comparison of trends across analytic methods. Individual relative trends are now displayed in **Figure 4**. We also have conducted a regression-based analysis to determine what disciplines exhibit a trend significantly different from that of the overall trend. For those disciplines that have a statistically significant deviation from the overall trend, we present those along with the overall trend in **Figure 4**. The details of the model are provided in the manuscript:

*“While baseline differences in the proportion of articles that mention an analytic method category are important to consider, of primary interest for this survey are the relative trends in mentions across the timespan of the study (2009-2020). To assess the statistical significance of the linear trends (i.e. decline or increase) in analytic method categories across the study timespan, we used a logistic regression model. Specifically, for each analytic method, we modeled the log odds of an article mentioning that method conditional on an article’s publication year (2009 - 2020) and the article’s scientific discipline. To assess whether any research discipline exhibits a statistically significant deviation from the overall linear trend of a given analytic method category, interactions between the linear trend and discipline were added to the model. To account for the correlated/non-independent structure of articles published within journals, the logistic regression model was estimated using a generalized estimating equations approach. Full details of the model are provided in the Methods and Materials section.”* (Pg. 8)

**7. In the section "Unique Method Usage by Discipline", the authors identified some method categories uniquely associated with each discipline. The word "unique" here is misleading because the same research method entity can still be present in multiple research disciplines.**

**Response:** We thank the author for noting the problem with this section title. There can indeed be the same entity identified across disciplines. We have now avoided this phrasing in the section title. Further, we have distinguished between those methods that appear across more than one discipline in the top 10, and those unique to a given discipline. The updated text in the manuscript:

*“Analytic Method Mentions by Discipline*

*To examine analytic methods associated with each BLS discipline, we used a contingency table approach. Specifically, we modeled the difference in observed versus expected article counts based on the number of article counts for each discipline and analytic method across the corpus. We used the standardized Pearson chi-square residuals as an effect size measure of the degree to which an analytic method is more prominently associated with a given discipline relative to other disciplines. In* ***Figure 5,*** *we display the top ten analytic methods per discipline as measured by the chi-square residual value.* *The raw probability for each analytic method by discipline is provided in Supplementary Materials (****S2 File)****.*

*As can be observed from* ***Figure 5****, each discipline is associated with a distinct set of analytic methods. Some analytic methods appear across more than one discipline - e.g. Fourier analysis in chemistry/material sciences and engineering/biotechnology. Others are unique to a given discipline.”*

**The authors used a contingency table approach (the Pearson chi-square standardized residual for each cell) to identify the associated the category. I am wondering if the approach is the same as the "revealed comparative advantage" [Trade liberalisation and "revealed" comparative advantage" The Manchester school 33, no. 2 (1965): 99-123], which is widely used in many fields such as [The product space conditions the development of nations." Science 317, no. 5837 (2007): 482-487]. Not sure if they are similar or the same but with different names.**

Response: We appreciate this observation. We are not deeply familiar with the ‘revealed comparative advantage’ approach that the reviewer refers to, however at first glance it does appear to be a similar approach.

**8. In Figure 6, the authors only presented parts of all 16 components. It would be better to present all of them, otherwise the reason of excluding some of them should be given.**

**Response:** We appreciate this suggestion. The 11 components of the 20 component solution were chosen to illustrate interesting patterns across disciplines in method groupings. We have now included a supplementary figure providing *all* components of the 20 component solution for the sake of transparency vis-a-vis future readers of our paper (**S5 Figure).**

**In the caption, the authors presented that "a stem plot illustrates the weights for each discipline, as well as the top 5 method entity strings". However, it seems that top 10 method entities were given, not top 5.**

**Response:** We thank the author for catching this mistake, the text has now been corrected.

**Moreover, the author presented "Some sets of methods are represented across all BLS disciplines (e.g., component 2), while others are concentrated within one or two disciplines (e.g., component 15)". Yet, this observation is very tricky because the scale of the x-axis (discipline weight) is very different. The authors are suggested to use the same scale for the x-axis.**

**Response:** We thank the author for providing the opportunity for us to clarify this important point. We would like to emphasize that the weights of the component have no ‘absolute’ meaning, and weights are only interpretable relative to each other. We present differing x-axes to illustrate the *relative ranking* of each method grouping across analytic methods. It is these rankings of disciplines within the stem plot that we believe are meaningful.

**9. In the Methods section (and the abstract section), the sample size is no longer a total of 3.5 million articles. Only 1,276,452 articles have the sections of Methods and Materials. I am wondering if this should be the starting point of the sample size.**

**Response:** We agree with the author that the original text was inaccurate. We have now included the number of articles in the corpus that went into our final analysis (after updating of the corpus with new data since our original submission). Now, our revised abstract reads:

*“Our survey of peer-reviewed literature analyzed ~1.3 million openly available research articles to monitor the cross-disciplinary mentions of analytic methods in the past decade.”*

**Moreover, it is not clear why the authors thought the PLOS ONE data is biased. It should not because it contained a disproportionate number of articles in the corpus. If so, all journal data is biased because they did not publish the same number of papers.**

Response: We initially downweighted PLOS ONE because the number of publications in the journal was an order of magnitude larger than the other target journals under consideration. However, in response to reviewer suggestions we have now included all PLOS ONE articles in our analysis.

**In addition, the total number of method categories resulting from our manual classification was 126. It is not clear the granularity of the classification and the quality of the grouping.**

**Response:** In addressing reviewer comments for the revision, we conducted a more thorough review of entities in our corpus during the disambiguation phase. The updated analysis now includes 250 manually classified entities. This classification was meant to be as granular as reasonably possible. However, we realize that any categorization is pragmatic, and open to interpretation. We have included our classification grouping of all entities on our Github page for transparency and reuse: <https://github.com/tsb46/stats_history/blob/master/analysis_main/ent_classification.csv>. We encourage interested readers to test their own hypotheses and entity classification systems which is seamless using this code.

**10. It seems that a majority of the Methods section can be relegated to the online supplementary information.**

**Response:** We appreciate this suggestion, and would be happy to move portions of the Methods to Supplementary Information at the request of the editor.

**In the reference section, some journals are abbreviated while others are not. The authors are suggested to use the same standard.**

**Response:** For the revision, all references have been updated and standardized according to the guidelines of Plos Biology.

**Reviewer #3:**

**Overall, the study addresses a very interesting and a significant practical issue concerning the use of research methods. Text mining techniques are applied to the methods sections in a collection of open access publications in biomedicine and some other disciplines.**

**Response:** Thank the reviewer for this enthusiastic, positive evaluation.

**PLOS Biology may be a good venue to publish the work, but I am wondering whether PLOS One would be more appropriate, given that the work would be accessible to other disciplines as well as biology.**

Response: We thank the reviewer for the suggestion for alternate avenues for this work. Given the interrelated research agendas of the biological, life and social science disciplines in the past decade, we think that the readers in the biological sciences are especially well positioned to benefit from a comprehensive survey. We ultimately leave it up to the editor to decide the suitability of this work for publication in PLOS Biology.

**The organization of the presentation is not straightforward. It should follow the logical flow, i.e., your question, your method, results, discussions and conclusions. Currently, it reads like a montage of various descriptions.**

**Response:** We appreciate the suggestion to improve the organization of our presentation. The entire text has been revised with an eye towards improving logical flow.

**What is commonly missing from the presentation is some minimal justifications of the choices you made, especially when multiple options are apparently available and even some of better alternatives are available as well. A large number of analytic techniques are used, MDS, tensor decomposition, CNN, Eucliedian-based similarity metric, etc. Is the wide variety of tools strengthening the study overall as opposed to using a small number of tools but each one digs deeper instead?**

Response: We appreciate the suggestion to provide greater justification for our analytic choices. Throughout, we have now included a brief rationale for each approach that was utilized, and described why we feel it is optimal for the question at hand.

**Some relatively minor issues are identified below:**

**The title is too complex and too long. Something like the short title would be much clearer.**

**Response:** We appreciate this point. We have revised the title to read: “The Changing Landscape of Analysis Methods in the Biological and Social Sciences”.

**Since the initial ~3.5 million was narrowed down to a subset and the methods section was the target, you should just mention the number of sections that satisfied your selection criteria.**

**Response:** We thank the reviewer for this suggestion. We now refer to the total number of articles that met our selection criteria (~1.3 million articles) in the revised text.

**Does the scope of BLS accurately reflect the study? It is not clear from the introduction.**

**Response:** We have now clarified the scope in the revised introduction.

**'computer- or qualitative-based assessment of measured data points'**

**how about:**

**quantitative and qualitative assessments of data.**

Response: We have implemented the suggestion of the reviewer:

*“ ‘Study’ is also broadly defined as a peer-reviewed quantitative- or qualitative-based assessment of measured data points”*

**The Introduction section is immediatedly followed by the Results section. Since many concepts and operations are not yet explained, it is too soon to present the results.**

**Response:**

We thank the reviewer for providing the opportunity for us to include more detail regarding key concepts germane to our study.

Our intention in the Introduction was to present the motivating questions behind the study and the approach to answer those questions (we have also added a brief summary of the main results in the new revision). The results section is intended to be read without first reading the ‘Materials and Methods’ section, as the ‘Materials and Methods’ section is placed at the end of the text in the manuscript. In other words, the results section is intended to be ‘self-contained’, allowing a reader to understand the results without first reading through a detailed methodology section. Thus, a high-level summary of the corpus and preprocessing pipeline are included at the beginning of the results section. Each section afterwards, contains a brief summary of the analysis approach implemented.

**What is described in the Results section is not really about results. Instead, they are the steps you take. More details**

**are necessary here. For example, "We used a named entity recognition algorithm trained specifically for this purpose."**

**What exactly does the algorithm do? What are the specific requirements to meet for this specific purpose? Similarly, a supervised machine learning framework was used. More details and justifications should be provided.**

**Response:** In line with our response to the previous comment, we have now included brief methodological details before each section for the reader to grasp the analysis approach without first reading through a detailed methodology section.

**To create 15 research disciplines, you extracted information from titles, abstracts and journal names, why not from keywords, which should be available from many of the articles and they are likely to improve the quality of your discipline identification and classification.**

**Response:** To clarify, we chose the title, abstract and journal names as the inputs to the classifier as this information was present across all articles in our corpus. Unfortunately, many articles that we surveyed in the corpus did not include keywords or phrases (as not all journals require them), so these were not included in the classifier model.

**Figure 1: step 4 is not numbered in the pipeline. Perhaps you should include an example of how article-level information is used for article classification.**

Response: Article-level information, such as the title, abstract and journal name are converted to features for the classifier by converting the tokenized text into 1-grams (e.g. ‘clinical’), 2-grams (‘clinical medicine’) and 3-grams (‘clinical medicine research’). These features are represented as count indicators: >= 1 if the 1-gram, 2-gram or 3-gram is mentioned once or more in the article, 0 otherwise. A detailed methodology of the classifier approach is presented in the methods and materials:

*“To classify articles in the corpus into the 15 BLS disciplines, we input a bag-of-words feature-set (1-gram, 2-gram and 3-gram tokens), generated from the article abstract, title, and journal title, to a multinomial naïve Bayes (MNB) classification algorithm. We utilized a version of the MNB algorithm that corrects for an unequal number of instances across categories* [*^31^*](https://www.zotero.org/google-docs/?wdWZxI)*, as was present in our corpus. To reduce the number of features input to the MNB algorithm and improve prediction accuracy we applied a chi-square feature selection approach. Specifically, we chose the top 15,000 features from the bag-of-words feature-set, after ranking the full set of features based on their chi-square statistic value with the 15 discipline categories. The chi-square statistic is a measure of dependence between two categorical variables, and in our case, provides an indication of the degree to which a text token* (e.g. 1-gram, 2-gram or 3-gram) is associated with differences in scientific disciplines*. The final MNB model for training involved 15,000 features, 15 discipline categories to be distinguished, and 1,470 training samples. We assessed the model accuracy using a repeated K-fold cross-validation approach ( N_Folds_ = 10, N_Repeats_ = 10).”* (Pg. 20)

**The first mention of Figure 1A and 1B, do you mean Figure 2A and 2B?**

**Response:** We thank the author for noting this mistake. The reference has now been corrected.

**"We deployed classical multidimensional scaling (MDS) ... (Figure 1B). You should give a brief justification of your choice of using MDS.**

Response: MDS was chosen as one of the most widely embraced low-rank visualization techniques used across disciplines and for many years (Hastie et al., 2001) to visualize the similarities between disciplines in their overall analytic method counts. While MDS is not the only existing approach for embedding similarities into a lower-dimensional space, it is a simple embedding approach that doesn’t require the tuning of hyperparameters that is required when utilizing more advanced approaches - e.g. t-SNE, ISOMAP, LLE. Our justification for the use of MDS is now provided in the revised text:

*“To provide a visual illustration of the similarity between disciplines in their overall analytic method counts, we deployed classical multidimensional scaling (MDS). MDS is a simple manifold learning technique that expresses each discipline’s total analytic method counts in a parsimonious two-dimensional space (****Figure 2B****). The distances between the disciplines in the resulting plot reflect the dissimilarity/similarity in total analytic method counts.”*

Hastie, T., Tibshirani, R., Friedman, J., 2001. The Elements of Statistical Learning. Springer Series in Statistics, Heidelberg, Germany.

**There are a few similar issues with the use of other methods and tools without a proper justification,**

**especially when multiple options are available, for example, tensor decomposition.**

**Response:** The analytic approaches used in the paper were designed to answer specific questions about trends in analytic method mentions across time or disciplines. For example, our analysis of analytic method groupings sought to answer how the co-occurence of analytic methods differs across disciplines. A natural way to express a co-occurrence matrix across a third grouping variable is in the form of a 3-dimensional tensor. Tensor decomposition allows us to simultaneously model both the co-occurence of analytic methods and how they vary across disciplines. We are not aware of another approach that would be appropriate for this purpose.

**-> hmm, this answer is not 100% convincing to me yet; can’t you say something along the lines how you have more than a2D matix, hit is why you need to work with tensors and not matrices, which is why PCA and similar is not enougk, which is why TensorDecomposition has suggested itself as a natural choice**

**Plots in Figure 3 are not on the same scale, although they all represent proportion, or percentage. For example, the Summary**

**Statistics on the upper left ranges from 0 to 0.60, whereas Analysis of Variance (ANOVA) next to its right ranges from 0**

**to 0.125.**

**Response:** We thank the author for noting the less intuitive use of different scales when visualizing analytic method trends. The original presentation of analytic method trends in **Figure 3** was meant to illustrate the relative trends across the study timespan. This made comparison across analytic methods trends difficult. Thus, we have included a new figure (**Figure 3**) with all analytic method trends displayed in the same plot, allowing for easier comparison of trends across analytic methods. Individual relative trends are now displayed in **Figure 4**. Further, we have cautioned readers regarding comparison of individual trends across analytic methods in the individual plots of **Figure 4**.

**Figure 5 is very interesting. Methods at various levels of scope and abstraction are presented together, e.g., meta**

**analysis and Z Score normalization.**

**Response:** We thank the reviewer for noting the issue of granularity and scope in our original manuscript. For the revision, we conducted a more thorough review of entities in our corpus during the disambiguation phase. We have now 250 manually classified entities. This classification was meant to be as granular as possible. However, we realize that any categorization is pragmatic, and open to interpretation. Importantly, the results (time trends, discipline-by-method contingency table analysis, and method grouping analysis) from this classification scheme were the same as what we observed with the classification scheme used in the original manuscript. Thus, we believe our results are likely to be fairly robust to the choice of classification schemes for analytic methods. We have included our classification grouping of all entities on our Github page: <https://github.com/tsb46/stats_history/blob/master/analysis_main/ent_classification.csv> and we encourage interested readers to experiment with their own proposed classification schemes.

**The Discussion section**

**'Either way, our finds reinforce the concern that statistics and research method education in the BLS science is falling**

**behind ...' It is not clear to me how you can draw this conclusion from your results since the education was never**

**part of the study.**

**Response:** We agree with the reviewer that this statement does not follow logically from our results. We have now removed the statement. The opening paragraph of the discussion now reads:

*“The data analytic landscape of the biological, life and social sciences (BLS) is growing increasingly complex. The available tools for data analysis have grown exponentially in the 20th century due to the increased accessibility of computational resources, open source software and Big Data. When learning to conduct data analysis, the scientist-in-training is faced with a steep hill to climb. To make this climb easier, graduate and undergraduate education must reflect the current practices and trends in data analysis. We offer an automated 12-year survey of ~1.3 million open research papers to characterize the data analytic landscape of the BLS sciences. This study aimed to provide a snapshot of the ongoing methodological shifts across a variety of scientific communities.”* (Pg. 15)

**goals of select research methods -> goals of selected research methods**

Response: We thank the reviewer for noting this grammatical issue. We have replaced the phrase, as suggested .

'**The appropriate context for each test is controversial among statisticians (33, 34).' It would be informative if you can**

**give us more information about the controversy so we can see the connection between the controversy and any patterns shown**

**in your results.**

**Response:** We have now provided more detail in the text:

*“For example, consider the predominant use of Fisher’s Exact Test in immunology vs. the chi-squared test in clinical research (****Figure 5****). Both are statistical significance tests of the association between two categorical variables. The appropriate context for each test is controversial among statisticians, but Fisher’s exact test is commonly recommended over the chi-squared test with small sample sizes (33, 34). Despite this controversy, our analysis indicates Fisher’s exact test is generally preferred over the chi-squared test in the field of immunology, and vice versa in clinical research. Thus, one might hypothesize that either 1) the discipline of immunology works with smaller sample sizes on average than the field of clinical research, or 2) these differences arose for sociological or historical factors.”* (Pg. 16)

**Methods**

**'To ensure ... not biased ..., we selected 2,625 random ...texts with tagged phrases ... and fed them to ... NER...' I**

**can see you may capture those that are similar to the 700 phrases with this process, but it is not clear how going**

**through this process will eliminate the bias if the 700 phrases are biased to begin with.**

**Response:** We thank the reviewer for bringing up this point. The original reference to ‘bias’ was a poor choice of words. The use of a fixed-phrase list to help train a named entity recognition (NER) is a commonly used technique to help automate the often time-consuming process of training data creation. Using automatically-annotated text derived from this fixed-phrase list allows the NER algorithm to learn the ‘context’ around mentions of analytic methods. By context, we mean the phrases and grammatical features that surround the mention of an analytic method. Learning of this context allows the NER algorithm to recognize those phrases and features that ‘signal’ the mention of analytic method. Thus, in-principle such an approach would allow the algorithm to generalize beyond the fixed-phrase list to analytic methods outside that list. In the new analysis for the revision we have expanded the fixed-phrase list to over 1100 phrases to better capture analytic methods that may have been missed in the original analysis. To ensure that our collection of phrases was not limited to the authors’ limited domain-knowledge, we also included analytic methods from the STATO ontology (<http://stato-ontology.org/>), an ontology of statistical methods, in our expanded phrase list.

**Discipline Similarity Analysis**

**The Euclidean distance: why did you choose this particular metric in this case? Is this the most appropriate metric to**

**use in this case?**

**Response:** MDS was a natural choice of method in our study (cf. above), which in turn relies on Euclidean distance to compute the similarity between two variables. To ensure scale-dependence did not affect our assessment of discipline-similarity, the count vectors were z-scored to eliminate scale-differences before computing Euclidean distance. This rationale is now included in the revised manuscript.

**Reviewer #4:**

**In this meta-research article, the authors propose that examining the frequency with which different statistical methods are used in the literature will allow scientists to tailor education in data analysis to the needs of students in particular field. They assess the statistical methods used in a large body of open access papers and conclude that use of conventional statistical methods is decreasing, while use of developing data science techniques is increasing. They further examine field specific patterns.**

Response: We thank the reviewer for this concise summary and helpful suggestions to further improve the manuscript.

**While the idea is compelling, the authors consistently rely on small differences over time or between disciplines when interpreting their data, without consideration of baseline values or the magnitude of observed changes/differences. This creates the impression that they are consistently making choices when presenting the data to show that more training in data science is needed, rather than interpreting the data as a whole in an unbiased manner.**

**Response:** We thank the reviewer for outlining this critique of our approach. We have taken several positive steps to address these legitimate concerns.

**Specific concerns are as follows:**

**1.	Figure 1 is quite difficult to interpret. In panel A, the abbreviations are not always intuitive, forcing the reader to switch back and forth between the figure and legend so frequently that the message is lost. The small font in the figure exacerbates this.**

Response: We thank the reviewer for pointing out remaining problems with Figure 1. We have now included a list of the disciplines and abbreviations in the main text so that they are introduced and familiar to the reader before the introduction of Figure 1. We have also increased the font size for all text in the figure to make it easier to read, as suggested. Here is the updated Figure 1:

*Figure 2.* ***Journal, Discipline and Analytic Method Statistics.*** *(BIOCHEM: Biochemistry/Cellular Biology/Molecular Genetics; CLINIC: Clinical/Hospital Research; CS: Computer Science/Informatics; CHEM: Chemistry/Material Science; ENVIRON: Environmental/Earth Science; ECO: Evolution/Ecology; IMMUN: Immunology; ONCO: Oncology; PSYCH: Psychology; NEURO: Neuroscience; EPIDEM: Public Health/Epidemiology; ENG: Engineering/Biotechnology; PHYSIO: Human Physiology/Surgery; POPGENE: Population Genetics; ANIMAL: Animal/Insect/Plant Sciences).* ***A)*** *A horizontal stacked bar plot displaying the number of articles for the top 20 journals in the corpus (defined in terms of article count). The percentage of articles per domain within a journal are proportionally shaded within each bar (IJERPH = International Journal of Environmental Research and Public Health). The research disciplines with the highest article counts were primarily biomedical and clinical disciplines.* ***B)*** *Multidimensional scaling (MDS) plot displaying the similarity between research disciplines, in terms of total analytic method counts (summed across all articles in the discipline), on a two-dimensional space. The x- and y-axis correspond to the two latent dimensions estimated from the MDS solution. The distance between two disciplines in this two-dimensional space communicates the dissimilarity in total analytic method counts between the two disciplines.* ***C)*** *Top 50 analytic method entities were ranked row-wise by the number of mentions across the corpus. The size of each entity string is proportional to the logged (log_10_) article count. The most frequently mentioned analytic methods were null hypothesis testing, correlation, confidence intervals and linear regression.*

**The figure is set up to show the proportion of papers in each journal that fall with in specific fields; yet many readers are likely more interested in what journals are represented in fields that are of interest to them.**

Response: We thank the reviewer for this suggestion. The intention of this figure was to communicate the largest journals (in terms of article counts) in our corpus. This information is important to understand the representation of journals in our corpus, and what journals may have outsized influence on the results over other smaller journals. We agree with the reviewer that the top journals by discipline would likely be a data point of interest. We have provided a table of the top 10 journals by discipline in **S1 File.**

**In panel B, there is no explanation of what the two dimensions represent.**

Response: We thank the reviewer for noting this omission from the text. We have now included a detailed explanation of what the two axes represent in the legend for Figure 2B:

***“B)*** *Multidimensional scaling (MDS) plot displaying the similarity between research disciplines, in terms of total analytic method counts (summed across all articles in the discipline), on a two-dimensional space. The x- and y-axis correspond to the two latent dimensions estimated from the MDS solution. The distance between two disciplines in this two-dimensional space communicates the dissimilarity in total analytic method counts between the two disciplines.”*

**The legend on circle size isn't tied to specific numbers of articles, and the circles in the legend are generally much larger than those in the graphs. The legend provides no information that would help one to interpret panel B.**

Response: We thank the reviewer for providing the opportunity for us to clarify. The legend was intended to simply illustrate that the size of each discipline’s node is proportional to their number of article counts, and does not provide exact quantitative numbers for each size. Thus, the node size in Figure 2B is meant to communicate relative differences between disciplines in their number of article counts. We agree with the reviewer that the exact numbers of article counts by discipline are useful data points.We have now included that information in a supplementary file (**S2 File**).

**In panel C, there is again no legend tying specific rows to entity counts. Readers are supposed to assess differences in counts based on font size, however this is not something that the human eye can accurately do.**

Response: We thank the reviewer for noting the limitations of Figure 2C. We agree that comparison of analytic methods by counts is difficult to do based purely on the font size of the text. Thus, the entity counts are ranked row-wise so that the reader can tell which analytic methods are greater than another by comparing their position in the figure. We have now made this clear in the figure legend text:

***“C)*** *Top 50 analytic method entities were ranked row-wise by the number of mentions across the corpus. The size of each entity string is proportional to the decimal logarithm of the (log_10_) article count.”*

We agree that exact differences in counts would be helpful to include.We have now included analytic method counts by year in a supplementary file (**S3 File**).

**Groupings based on conceptual similarities between different entities are discussed in the text, but do not appear in the figure. This figure needs to be redesigned to convey a clear message.**

**Response:** We thank the reviewer for allowing us to clarify our categorization process of analytic methods. In the final stage of pre-processing, the entity strings from the NER algorithm (after text preprocessing) undergo a manual entity disambiguation process. In this process, the authors categorized together entity strings that refer to an identical analytic method as the same entity. For example, the entity strings ‘Cox PH regression’, ‘Proportional Hazards regression’, and ‘Cox Regression’ were classified as the same analytic method. This step was to ensure that references to the same method with different titles did not affect our results. The analytic method phrases shown in Figure 2C display the analytic methods from this process. When analyzing trends over time, we grouped analytic methods from the entity disambiguation phase into larger conceptual (superordinate) categories (e.g. ‘ANOVA/T-test’, ‘Survival Analysis’, etc.) to analyze large-scale trends in conceptually similar analytic methods.

**2.	The authors choose the term "method entities" to refer to statistical analysis methods throughout the paper. Given that their focus is on analytic methods, a more specific term may be beneficial. Many readers will assume that method entities refer to the complete range of laboratory and clinical methods used in their field.**

Response: We agree that this term is misleading. We have now replaced all references of ‘research method’ or ‘method entities’ with ‘analytic methods’ to make our object of study more clear.

**3.	Figures 3, 4: The legend of figure 3 suggests that the top panel is missing.**

Response: We thank the reviewer for noting this error, and have corrected the text.

**Both figures use small multiples; yet the authors have violated a key rule of small multiples by using completely different scales in each graph. This makes it impossible to tell which methods are common vs. uncommon, and which increases/decreases are small vs. large. The authors have done this because some of the percentages and increases are so small that they would not be visible on a uniform scale. One has to look closely to see that many of the machine learning categories, for example, are used in less than 1% of papers. In contrast, the traditional method categories are generally used in 10-15% of papers. These observations illustrate the danger of drawing conclusions based solely on increasing or decreasing patterns, without reference to baseline values or the magnitude of the observed changes.**

**Response:** We thank the reviewer for noting this problem with our interpretation of analytic method trends in the original manuscript. We have now included a new figure (**Figure 3**) that displays all analytic method trends on the same plot. This figure shows that baseline differences in analytic method mentions are an important source of information when comparing trends. We see that statistical methods commonly taught in introductory research methods and statistics courses are the most commonly mentioned methods across the corpus. We have now highlighted the importance of these baseline differences in the text at various points:

In interpretation of Figure 3:

*“As can be observed from* ***Figure 3****, introductory statistical methods - e.g. null-hypothesis, testing, t-tests/ANOVA, linear regression, confidence intervals - have remained the dominant analytic methods mentioned in BLS methodology and results sections over the past decade. Null-hypothesis testing concepts and statistics (e.g. p-values, null hypothesis, and alternative hypothesis) are by far the most commonly mentioned analytic ‘methods’ in the BLS sciences - approximately 35% of articles per year mention one or more analytic methods in this category. The next most prominently mentioned analytic method category are ANOVA/t-tests - statistics for comparing mean differences between one or more means collected from independent groups or repeated observations. Interval estimation approaches (e.g. confidence interval and credible interval) for calculating the possible values of a population parameter is the third most prominent analytic method category.”*

In the discussion:

*“This study aimed to provide a sociological snapshot of the ongoing methodological shifts in a variety of scientific communities. We find that the analytic methods commonly taught in introductory research methods and statistics courses remain the most commonly mentioned methods in research article ‘Methods’ and ‘Results’ sections.”*

However, while baseline differences are important, a primary aim of this study was to examine relative trends in analytic method mentions across the timespan - are analytic method mentions increasing or decreasing over time. The use of a uniform scale precluded visualization of these relative trends for methods with smaller baseline counts. Thus, we have included individual trends in **Figure 4**, but provide the reader with a word of caution when comparing individual trends across analytic methods:

*“While baseline differences in the proportion of articles that mention an analytic method category are important, of primary interest for this survey are the relative trends in mentions across the timespan of the study (2009-2020). To assess the statistical significance of the linear trends (i.e. positive or negative trend) in analytic method categories across the study timespan, we fit a logistic regression model to each analytic method category to estimate the linear/decrease in the odds of an article mentioning methods of that category over the past decade. To assess whether any research discipline exhibits a statistically significant deviation from the overall linear trend of a given analytic method category, interactions between the linear trend and discipline were included in the model. To account for the correlated/non-independent structure of articles published within journals, the logistic regression model was estimated using a generalized estimating equations approach. Full details of the model are provided in the Methods and Materials section.*

*The trends in analytic method category mentions are plotted individually (plotted in blue) in Figure 4****.*** *Note, y-axis scales are relative for each analytic method category and caution should be taken in comparing trends across categories****.”***

**4.	Similar concerns apply to figure 5 - the data presented focuses on differences between observed and expected, without consideration of how frequently each method is used within the given fields. The authors' hypothesis is that examining the frequency with which methods are used, and patterns of change, will allow us to tailor education within different disciplines. Yet, readers cannot use this figure for this purpose, as it doesn't consider the frequency with which methods are used. A data driven summary figure showing commonly used methods in each field, in addition to emerging techniques in which students would benefit from additional training, may be beneficial.**

Response: We thank the reviewer for noting similar problems with Figure 5. The intention was to communicate those analytic methods with higher-than-expected mentions in a given discipline. We agree with the reviewer that this information would be helpful to provide. We have included a supplementary file (**S5 File**) with the proportion of articles mentioning each analytic method, for each discipline.

**5.	Validation and performance characteristics for the different methods used is lacking from the material and methods section. The authors describe how things were done, but they don't specify how well the algorithms work compared to gold standard ratings.**

Response: We thank the reviewer for allowing us to elaborate on the methods employed in the manuscript and their validation. Many analyses performed: multidimensional scaling (MDS; Figure 2B), generalized estimating equations for analytic method trends (Figure 4), the contingency table analysis for discipline-specific analytic methods (Figure 5) and tensor decomposition (Figure 6) lack universally accepted validation metrics. Overall fit statistics can be provided for these methods. For example, one can compute the Kruskal’s stress metric for MDS - in our case, our two-dimensional solution had an overall stress value of 0.21. For analysis methods with labeled data, such as the journal classification, accuracy metrics can be computed and are provided in the methods and materials:

*“We assessed the model accuracy using a repeated K-fold cross-validation approach (#folds = 10, # of repeats = 10). The final MNB model achieved an accuracy of 74.9%. The accuracy achieved for each individual discipline are as follows: ANIMAL – 71.2%, BIOCHEM – 72.2%, CHEM – 88.4%, CLINIC – 77.6%, CS – 79.2%, ECO – 75.3%, ENG – 67.4%, ENVIRON – 67.8%, EPIDEM – 65.6%, IMMUN – 64.0%, NEURO – 87.1%, ONCO – 84.5%, PHYSIO – 69.6%, POPGENE - 60.0%, and PSYCH – 69.2%.*”

We are unaware of any gold-standard accuracy criteria in text-based classification of research articles into select research disciplines.

The output of the NER algorithm was checked manually during the entity disambiguation phase and ‘false positive’ entity strings - phrases not referring to an analytic method - were thrown out. All entity strings detected by the NER algorithm after preprocessing that went into the entity disambiguation phase are provided in <https://github.com/tsb46/stats_history/blob/master/analysis_main/ent_classification.csv>.

Further, we have made all analyses and metadata publicly available for those readers who wish to conduct further queries : https://github.com/tsb46/stats_history.

**6.	While the article includes a limitations section, it is limited in scope. Authors may be more likely to report details of new or complex methods, compared to commonly used methods like t-tests and ANOVA, which would lead to undercounting and bias. Statistical reporting is poor in many fields. The authors may be able to incorporate checks into their data to assess the frequency of completely inadequate reporting (i.e. p-values reported, but no methods that generate p-values are listed).**

**Response:** We thank the reviewer for highlighting these important limitations. The limitations section has now been significantly expanded. In addition, we include in this section the reviewers concerns:

*“It should be noted that the corpus and methods used in this analysis are limited in many respects. First and foremost, our study relies on mentions of an analytic method in a research article as an indicator for the usage of that analytic method in the article. However, the mention of an analytic method is not an unequivocal indication of its usage for data analysis in a research article. We sought to decrease the chance of false positives by restricting our analysis of research articles to their ‘Methods’ and ‘Results’ sections, where mentions of an analytic method would be more closely associated with their usage. However, due to the infeasibility of spot-checking a large corpus (~1.3 million articles), there are undoubtedly false positive cases in our sample. Further, the entity recognition approach in our study requires that analytic methods are explicitly reported in methodology and results sections. However, inadequate and/or inaccurate reporting of protocols and statistical analyses is a known problem in the BLS sciences* [*^25–27^*](https://www.zotero.org/google-docs/?nPAiHH)*. Thus, there are likely a number of research articles in our corpus where the full set of analytic methods employed was not captured by our analysis.”*

**7.	The authors approach examines the frequency with which different methods are used; it cannot assess whether authors are using the appropriate method for the data that they are analyzing. It is likely that there is additional need for newer methods in many fields; yet uptake is limited by a lack of expertise. Differences in expertise and training for new methods across fields may also contribute to limited uptake in some fields compared to others, magnifying the field specific differences that the authors observe. Using data like those generated by the authors to set educational priorities could potentially exacerbate these problems. While data driven approaches are beneficial, one should not lose site of the fact that some of the excitement around these approaches is based on the potential of what could be achieved if they were well implemented on a broader scale. It may be too early to capture this using the approach described by the authors. Furthermore, a clear**

**discussion of what poor vs. good implementation looks like is needed - a proliferation of poorly conducted studies using the latest popular methodology has hindered progress in many fields.**

**Response:** We thank the author for highlighting this limitation of our approach. We have included the following text in our limitations section:

“Second, the usage of analytic methods does not imply that the method was applied appropriately or correctly. In fact, the inappropriate application of statistical methods may be a contributor to the replication crisis in the BLS sciences [^28–30^](https://www.zotero.org/google-docs/?HX2BjH) **.** Thus, the increase in usage of multivariate and machine learning methods should not be considered prima facie evidence that these methods are being used appropriately.”
