# Supplementary material for: Educating the future generation of researchers: A cross-disciplinary survey of trends in analysis methods
Source: PLoS Biol. 2021 Jul 29;19(7):e3001313. doi: 10.1371/journal.pbio.3001313 (PMC8321514; doi:10.1371/journal.pbio.3001313)
Supplement: Attachment — Submitted filename: Reviewer_Response_052321.docx [file pbio.3001313.s011.docx]

May 31, 2021

**Editor**

**a) Please address the remaining requests from the reviewers, including the additional robustness checks for rev#3 and the presentational requests from rev #4.**

**Response:** All remaining requests from the reviewers have been addressed.

**b) The current title is fine, but it might be more appealing to readers if it mentions (in a few words) the implications of your findings, e.g. for education of future generations of biologists.**

**Response:** We thank the editor for this suggestion. We suggest the following revised title:

Educating the Future Generation of Researchers - A Cross-Disciplinary Survey of Trends in Analysis Methods

**c) Please address my Data Policy requests below; specifically, please supply numerical values underlying Figs 2ABC, 3, 4, 5, 6, S1AB, S2AB, S3, and cite the location of the data clearly in each relevant Fig legend.**

**Response:** The numerical values behind all figures are now provided as supplementary material, according to the data availability instruction on the PLOS Biology webpage. Further, the location of the numerical values for each figure are now cited in their legends.

**d) Please supply a blurb, following the instructions in the submission form.**

**Response:** We have now created a blurb for the article.

“A quantitative survey of >1 million published research articles revealed that classical statistical methods remain in wide use in biomedical, life science and social science . However, use of these methods have declined over the past decade. Instead, multivariate statistical and machine-learning approaches have seen a significant increase in the total share of scientific publications. Revision of statistics curricula is needed to take full advantage of these new analytical tools.”

**e) Your financial declaration currently says that you had no specific funding for this work; can you just confirm that this is correct, or add appropriate funding information?**

**Response:** The primary author had no specific funding for the work, but three of the co-authors do have funding information which has now been provided in the acknowledgements and the online submission form.

**Reviewer 1**

**The authors have addressed all my points.**

**Response:** We thank the reviewer for their insightful suggestions and comments that greatly improved our manuscript.

**As the new figure with the clustermap is referred to as Supplemental Figure 2 within their reply, but as Supplemental Figure 1 within the manuscript, they might verify that the placing of this Supplemental Figure within the manuscript is as intended.**

**Response:** We thank the reviewer for noting this mistaken reference within our reply. We have ensured that the figure is correctly mentioned in the manuscript as Supplementary Figure 1 as intended.

**Reviewer 2**

**Review report for "The Changing Landscape of Analysis Methods in the Biological and Social Sciences" by Bolt et al.**

**I would like to thank the authors for considering my comments and suggestions on the previous version of the paper. In my view, the authors have tried to improve their work and they did a nice job. I am largely satisfied with their responses to my comments. In this round, I only have a few minor comments which may be helpful for the authors to further strength their paper.**

**Response:**

**Response:** We thank the reviewer for the kind words, and we appreciate their additional suggestions to further improve the manuscript.

**The authors argued that the number of references to the same analytic method in a paper does not provide evidence for the frequency of its usage in the paper. Also, it is possible that some entities identified by the NER algorithm were not actually used in the paper. I am wondering what the results would look like if the authors do a weighted version of entities, i.e., the intensity of references to an analytic method by a research discipline. Maybe include some robustness checks in the supplementary.**

**Response:** We thank the reviewer for their suggestion. As mentioned by the reviewer, we argue that the number of references to the same method does not provide robust evidence for its frequency of usage in a research article. Thus, the number of times a method appears in the same article was ignored. As a robustness check, we followed the reviewer’s suggestion to examine the intensity of references to analytic methods by research discipline. We conducted the analytic method by discipline probability analysis (**Figure 5**) including the frequency counts of each analytic method within an article . As shown below, this led to essentially the same results and the identical scientific conclusions:

In comparison with the original analysis (Figure 3), the exact position of some analytic methods in the top 10 for each discipline changed only slightly with the weighted version of the analysis, but the overall results remain the same. In other words, the top 10 analytic methods for each discipline is consistent across both methods.

**2. The authors acknowledged that one paper can belong to multiple research disciplines, for simplicity here each paper was categorized into the dominant discipline. They have included this simplification as a limitation in the discussion section. Still, I am wondering if the multi-classification of papers would have a significant impact in the results considering the increasing trend of interdisciplinary papers especially in recent years. Maybe include some robustness checks in the supplementary.**

**Response:** We thank the reviewer for allowing us to clarify the logic behind our discipline classification approach. Our classification algorithm was trained by a single discipline to article classification training dataset. Further, the accuracy of our model was tested against a single discipline to article classification testing sample. Given this training and testing classification scheme, it would be difficult to assess the true performance of our classification algorithm against a multi-labeled testing sample, as the algorithm was not trained to perform multi-label classification. However, we have provided the original Python code behind the classification algorithm for those who wish to create a multi-label classification training and testing dataset for future research.

**3. Besides the mention of a single method by different disciplines, it may be also interesting to consider the co-occurrence of two method entities across disciplines, or a group of entries that are co-occurred very often. In my view, this is another promising direction for future studies, and this can be mentioned in the discussion section.**

**Response:** We thank the author for bringing up this important point. In fact, the goal of the ‘analytic method groupings’ analysis was to understand method co-occurrences across disciplines (**Figure 6**). This method utilized tensor decomposition of a discipline by method*method co-occurrence matrices to directly estimate those groups of methods that tend to co-occur together, and how their co-occurrence varies across disciplines. For each component, the analytic method weights reflect groups of analytic methods that tend to co-occur together, and the discipline weights reflect the disciplines where these method co-occurrences are most common.

**4. I found the first few sentences of the abstract and the first paragraph of the introduction particularly interesting, considering that the changing use of methods across disciplines may lead to educational reform to undergraduate and graduate quantitative research method curricula. It may be good to echo these broad conservations in the discussion sections and suggest more policy implications of the findings in this paper.**

**Response:** We thank the reviewer for highlighting the absence of these important points in the discussion. We have now included a brief discussion around the current state of statistical education and our suggestions for a more ‘data-science’ focused curriculum:

*“Projecting these trends into the future, we would expect that multivariate statistics and machine learning methods will enjoy increasing usage relative to more traditional statistical testing frameworks. The traditional statistical tool stack - t-test, ANOVA, z-test, etc. - taught in undergraduate and graduate statistics courses were largely developed in the first half of the 20th century*[*^32^*](https://www.zotero.org/google-docs/?8MJgPu)*. Since then, advances in computation and statistical computing software have revolutionized the analytic tools available to researchers. The application of advanced multivariate and machine learning analysis methods requires proficiency in statistical software and/or open source programming languages that are largely absent from most traditional research methods and statistics curricula. Overall, these trends suggest that introductory research methods and statistics courses may benefit from incorporating a ‘data science’ focus in their curricula* [*^8,32^*](https://www.zotero.org/google-docs/?aYwxGd)*.”* (Pg. 15-16)

**5. Some minor suggestions on figures:**

**(a) The x-axis in Figure 2A can be round to "int".**

**Response:** The x-axis has been rounded to remove the decimal notation.

**(b) The legend of Figure 3 can be sorted by the result of the latest year 2020. This alignment will better guide the eyes of readers.**

**Response:** We thank the author for the suggestion to improve the readability of the Figure 3 legend. We have now sorted the methods in the legend by the proportion value of the last year (2020). In addition, we have followed the suggestion of Reviewer 4 to denote the end of each line (time series) with a method label along with the legend.

**(c) The resolution of Figure 4 should be largely enhanced. In addition, the legends in some panels are much overlapped with the curves. May consider a two-column legend layout when it helps.**

**Response:** The figure has now been converted to a vectorized .pdf format to significantly improve the resolution of Figure 4. The legend in each graph has been made more transparent to allow lines to be observed behind the legend box.

**Overall, the authors did a great job. Good luck with the submission and publication.**

**Response:** We thank the author for the kind words, and are grateful for their insightful suggestions to significantly improve the manuscript.

**Reviewer #4:**

**Fig 3 - The legend has many colors; it will be difficult to make the color palette accessible to colorblind readers, and even readers with normal color vision will need to search through and color match to find what they are looking for. To enhance readability, the authors might consider labeling each line on the right hand side (where the legend is) next to the line. This would allow readers to quickly scan down and determine which methods are most common. Colored lines from the legends could still be used for cases when different lines are close together.**

**Response:** We thank the reviewer for their suggestion on how to make the figure more legible. We have added labels to the end of each line (to the right) to reduce the need to look back and forth to the legend. In addition, we have followed the suggestion of Reviewer 2 to order the method labels in the legend according to the proportion value of the last year (2020). These changes have significantly improved the readability of the figure.

**Fig 4 - The authors may want to consider providing a "y-axis location" legend; a small box or rectangle next to the legend of each figure sized so that the y-axis runs from the minimum to the maximum of any panel shown (0 to 0.65), on which the region encompassing the scale for the y-axis of the particular graph is highlighted. This would give readers a quick visual indicator to help with comparing across graphs. (For example, for NHST, the region from 0 to 0.25 would not be highlighted, whereas the region from 0.25 to 0.65 would be highlighted. For PLS/DA, the region from 0 to 0.02 would be highlighted).**

**Response:** We thank the reviewer for proposing this creative solution to the problem of differing y-axis scales across graphs in Figure 4. We have followed the reviewer's suggestion and added a shaded rectangle to the bottom right of each figure to illustrate the range of the y-axis scale (from the minimum to maximum possible proportion value across graphs).
